# The influence of teriparatide in induced tooth movement: A systematic review

**DOI:** 10.4317/jced.52997

**Published:** 2016-12-01

**Authors:** Bianca-Núbia Souza-Silva, José-Lucas-Sani-de Alcântara Rodrigues, Jefferson-Chaves Moreira, Felipe-de Souza Matos, Carla-Patrícia-Hernandez-Alves-Ribeiro Cesar, Carlos-Eduardo-Palanch Repeke, Luiz-Renato Paranhos

**Affiliations:** 1 Undergraduate student, Department of Dentistry, Federal University of Sergipe, Lagarto, SE, Brazil; 2 DDS, MSc, Postgraduate Program in Dentistry, Federal University of Sergipe, Aracaju, SE, Brazil; 3 DDS, MSc, PhD, Professor, Department of Speech Therapy, Federal University of Sergipe, Lagarto, SE, Brazil; 4 DDS, MSc, PhD, Professor, Department of Dentistry, Federal University of Sergipe, Lagarto, SE, Brazil

## Abstract

**Background:**

Teriparatide is a synthetic drug similar than PTH (parathyroid hormone), which is currently used as long-term treatment option for patients with bone chronic diseases, as osteoporosis; and this drug can interfere in a positive way in orthodontic movement. Objectives: The medical literature was assessed in the present systematic review in order to determine the level of scientific evidence supporting the influence of teriparatide in induced tooth movement.

**Material and Methods:**

The PRISMA Checklist was followed in this systematic review. Four electronic databases (PubMed; Scopus; ScienceDirect; OpenGrey) were searched without implementing restrictions of year, status, and language of publications. The inclusion criteria consisted of selecting only experimental studies comparing the influence of teriparatide in tooth movement of male Wistar rats. The exclusion criteria consisted of experiments with female rats or other experimental animals, and animals with pathologic conditions. The eligible studies were evaluated based on methodological quality. Two trained examiners performed all the research steps.

**Results:**

The initial sample comprised 700 studies, which was reduced to 664 after the exclusion of duplicates (n=36). Three articles were selected for the final qualitative analysis. The local administration of parathyroid hormone (PTH) 1-34 or PTH 1-84 revealed major effectiveness when compared with control groups and systematic administration. Additionally, the dilution of PTH 1-34 within methyl cellulose (MC) gel increased the time range for drug release, enabling to reduce the drug concentration without decreasing the effectiveness of tooth movement.

**Conclusions:**

Teriparatide demonstrated potential acceleration of tooth movement in Wistar rats depending on the drug concentration; drug administration; and time for drug release.

** Key words:**Teriparatide, tooth movement, parathyroid hormone, orthodontics.

## Introduction

Life expectation considerably increased in the last decades, changing social lifestyle and personal health habits. Consequently, adult patients became more aware of oral health care, boosting the demand for orthodontic treatment. In this way, orthodontic clinics started treating a population of patients with heterogeneous age range. In addition, these patients have different genetic profile and health status, which potentially interfere within the time and efficiency of tooth movement (1,2).

Basically, orthodontic movement is founded on the application of vector forces in the teeth, generating two opposite responses in different areas of periodontal ligament. The first consists of pressure, which we observe an aseptic inflammatory response triggering the activation of osteoclasts and bone resorption. The second consists of tension, which is generated an anti-inflammatory environment conducive to activation of osteoblasts and bone formation (2-4).

Therefore, systemic bone diseases may interfere within orthodontic treatment culminating in adverse biological response during tooth movement, directly influencing the treatment outcomes. Osteoporosis emerges as one of the most prevalent bone diseases in adults, affecting more than 75 million people worldwide, causing the reduction of bone density due to excessive osteoclastic activity (5). Currently, the long-term administration of teriparatide, a synthetic form of PTH, in low doses represents one of the treatment options for osteoporosis. Paradoxically, the same PTH, which in high concentrations may culminate within osteoporosis, may also enhance the longevity of osteoblasts, increasing the bone density through the elevation of bone turnover (6-9).

The hypothesis that improved bone responses, and possibly tooth movement, are achieved in patients undergoing treatment with Teriparatide was raised (10-12) resulting in controversial opinions among researchers in the field. Thus, the present systematic review aims to analyze the influence of teriparatide administration in the induced tooth movement.

## Material and Methods

- Protocol and registration:

This systematic review was done adhering to the Preferred Reporting Items for Systematic Reviews and Meta-Analyses PRISMA (13) checklist (www.prisma-statement.org). We did not register a protocol.

- Focused question:

The present research aimed to answer the following guiding question: Is the teriparatide administration optimally influencing induced tooth movement?

The research question was based on the PVO strategy for Systematic Exploratory Review, where P stands for population, context, and/or problem-situation, V stands for variables, and O stands for desirable or undesirable outcomes.

- Eligibility criteria:

• Inclusion criteria: Experimental studies comparing the influence of teriparatide on tooth movement in Wistar rats. With no restrictions on year, publication status, or language.

• Exclusion criteria: Articles that investigated the teriparatide effect on another experimental animal; female rats; and animals with pathologic conditions.

- Information sources:

 A systematic search was performed in PubMed®, Scopus®, ScienceDirect® and OpenGrey® electronic databases. The data base OpenGrey® was used to check the gray literature and avoid selection bias.

- Search:

Medical Subject Headings (MeSH) and boolean operators (“OR” / “AND”) were used to build a search string with the terms “Teriparatide”, “Orthodontic” and “Parathyroid Hormone”. The search strategy ( Table 1) was performed in May 14th 2015.

**Table 1:**
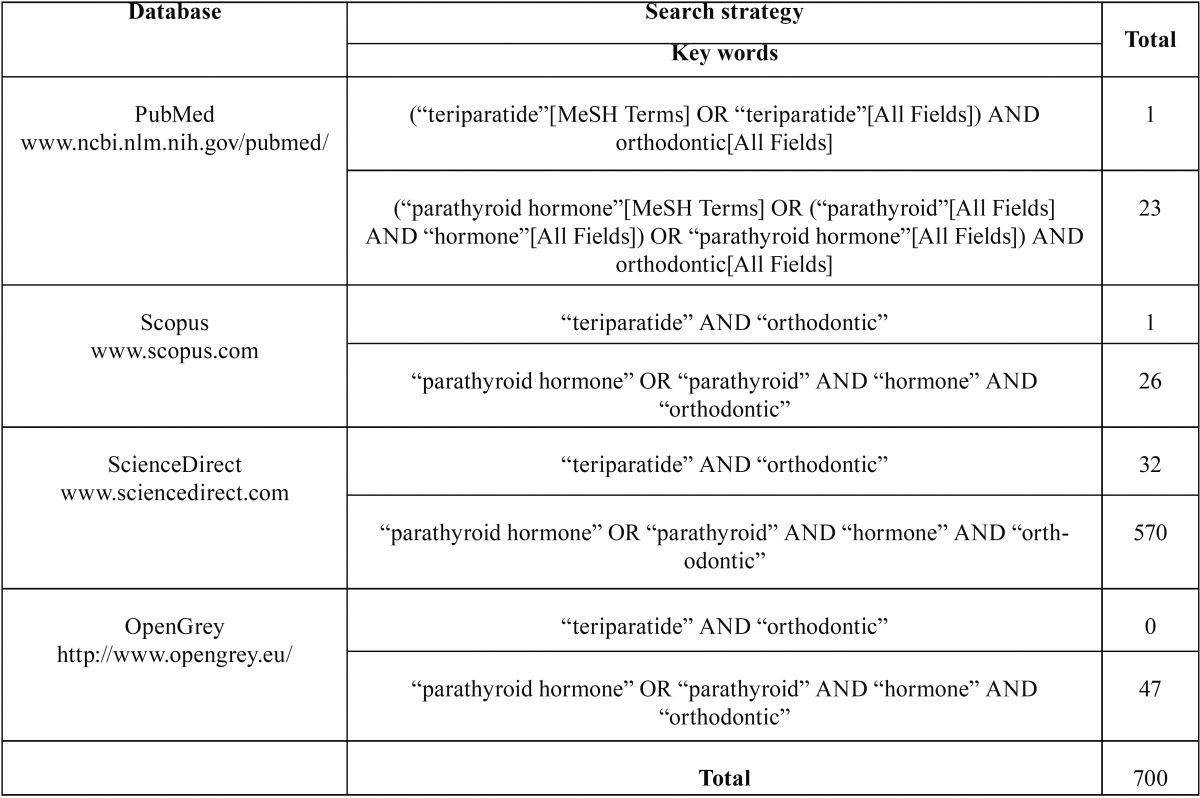
Electronic databases and applied search strategy.

 The results obtained were exported to the software Mendeley Desktop 1.13.3 (Mendeley™ Ltd, London, UK), where duplicity was verified.

- Study selection.

Selection was completed in 2 phases. Titles and abstracts were systematically assessed by two reviewers for eligibility (JLSAR and BNSS), and they were not blind to authors or journals. Whenever the title and abstract of articles did not present enough information, full texts were obtained and analyzed to decide on their eligibility. Articles presenting a title that fit the theme but with no available abstracts, were also obtained and fully analyzed.

The full texts of previously eligible articles were downloaded and read so to verify the presence of every inclusion criteria. In specific cases, the authors of potentially eligible articles were contacted by e-mail and asked for missing information. The rejected articles were registered separately, displaying the reasons for exclusion.

- Data collection process.

Two examiners assessed the risk of bias and quality in individual studies (JLSAR and BNSS). In case of disagreement, a third examiner (LRP) was consulted. At this point, the review was blindly performed, masking the names of authors and journals, avoiding any potential bias and conflicts of interests during sample selection.

- Data items:

After triage, the full texts of pre-selected articles were re-examined and their data were extracted in a standardized manner. The information extracted and recorded from the articles were authorship; year and place of publication; sample profile; orthodontic technique; drug administration; tooth movement rate in experimental and control groups; and clinical outcome.

- Risk of bias in individual studies:

The selected studies assessed the risk of bias and quality in individual studies using a standard checklist ( Table 2). Each study received a score adapted from Cericato *et al.* (14): low quality study (from 0 to 5 points); average quality (from 6 to 9 points); high quality (from 10 to 12 points).

**Table 2:**
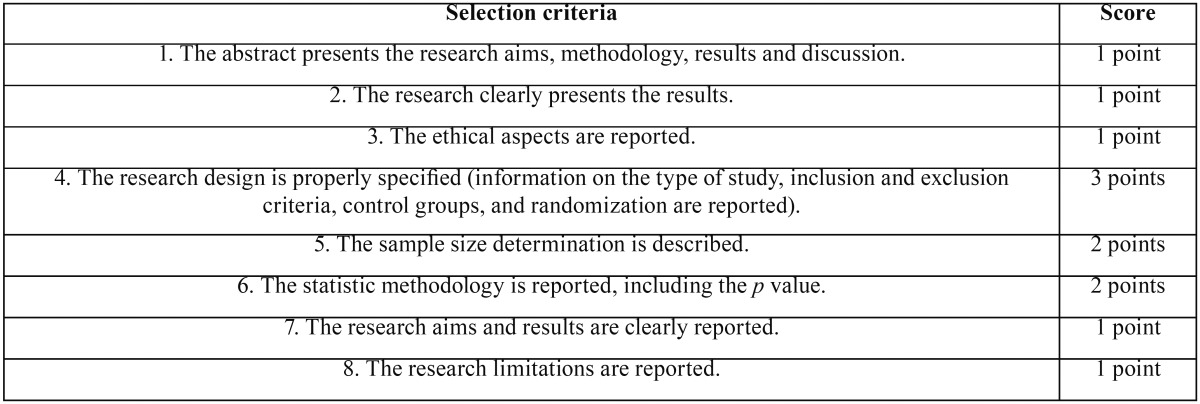
Scores and selection criteria adapted from Cericato *et al.* (14).

- Summary measures:

The process of data synthesis was performed through a descriptive analysis of the selected studies, and the final product of the analysis was presented in narration/dissertation form.

- Synthesis of results:

The process of data synthesis was performed through a descriptive analysis of the selected studies, and the final product of the analysis was presented in narration/dissertation form. A meta-analysis was planned, since the data from the included studies was considered relatively homogeneous.

- Risk of bias across studies:

Only to be applied if meta-analysis was possible.

## Results

**Figure 1:**
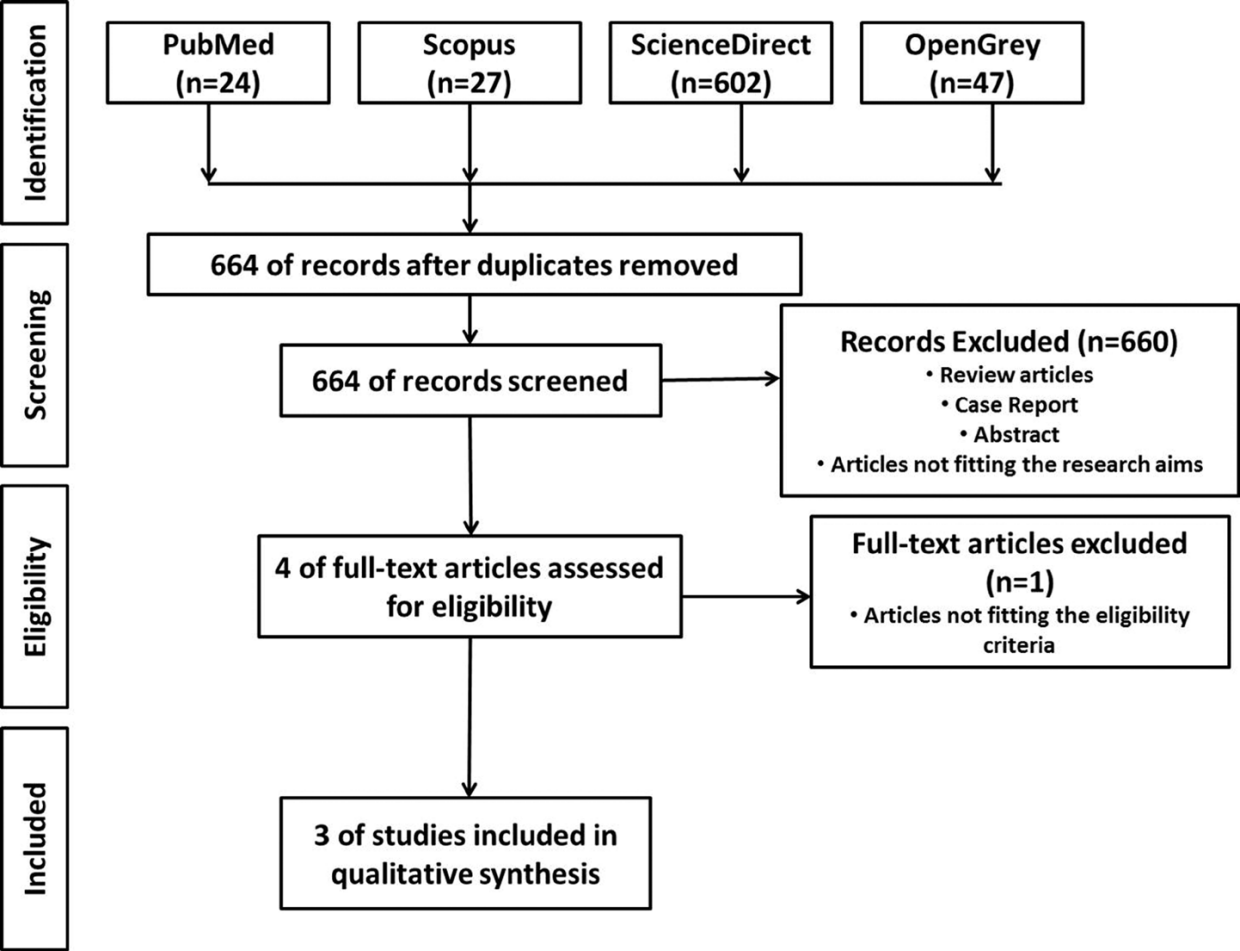
Flow diagram of literature search and selection criteria adapted from PRISMA.

- Study selection:

A flowchart describing the process of identification, inclusion, and exclusion of studies is shown in figure 1. A total of 700 results were retrieved during the first selection phase; after removing duplicates, the total was 664 results. After applying inclusion and exclusion criteria, three articles remained eligible.

- Study characteristics:

Two eligible studies (10,11) were published in Japan, while one Li *et al.* (12) was published in China. All the studies were published in English language between 1999 and 2013.

- Risk of bias within studies:

All the studies reached at least average scores based on previous evaluation ( Table 2). Some aspects became specifically evident, such as the lack of sample size calculation in the three eligible articles, and the lack of discussion on the study limitations in one eligible article (11).

- Results of individual studies:

Soma *et al.* (11) injected subcutaneous PTH 1-34 and PTH 1-84 in 112 Wistar rats, in a daily basis, with concentration varying between 0.4-4.0 µg/100 g (PTH 1-34) and 10 µg/100 g (PTH 1-84). A second group received PTH 1-34 with concentration of 4.0 µg/100 g on the dorsal region of the animal. Control group did not receive PTH ( Table 3).

**Table 3:**
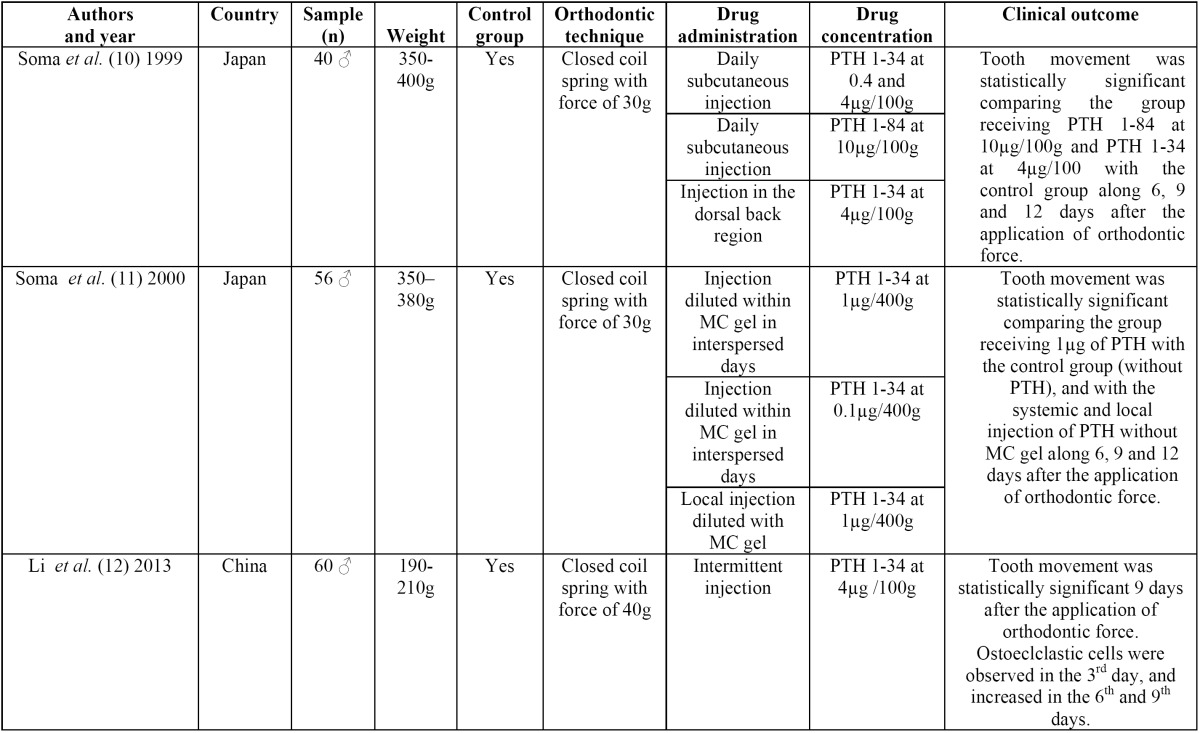
Summary of study descriptive characteristics of included studies.

Soma *et al.* (11) injected PTH 1-134 diluted within methyl cellulose gel 2%w/v in the mesiopalatal region of the maxillary first molar of 56 Wistar rates, with interspersed applications (every other day). Additional applications consisted of local injections of non-diluted PTH 1-34; and subcutaneous injection of PTH 1-34 concentrated in 1.0 µg/400 g on the dorsal region of the animal. All the animals underwent orthodontic force of 30g ( Table 3).

Li *et al.* (12) injected subcutaneous PTH 1-34 diluted within a buffered phosphate solution in 60 Wistar rats, with concentration of 4.0 µg/100 g. A control group was treated without the injection of PTH. All the rats underwent orthodontic force of 40 g ( Table 3).

In all the studies PTH 1-84 or PTH 1-34, also known as teriparatide, was injected in the moment “0” (day 0), and the closed coil spring was installed between the maxillary right first molar and the incisors. The activation of these orthodontic appliances was performed immediately after installation (day 1). No further reactivation was performed (10-12). The Wistar rats underwent euthanasia in experiment day 0 (before orthodontic movement), 3, 6, 9, 12 (10-12). Yet Soma *et al.* (11) designed a triple measurement methodology performed by three blind examiners ( Table 3).

- Synthesis of results:

Soma *et al.* (10) examined the tooth movement using a microscope at a magnification of 40 x aided by calipers with precision of 0.05mm.11 Similarly, Soma *et al.* (11) and Li *et al.* (12), used digital calipers with precision of 0.05mm and 0.02mm respectively.

Soma *et al.* (10) revealed that daily injections of subcutaneous PTH 1-84 with concentration of 1.3 and 10 µg/100 g significantly increased the number of osteoclasts in the region of pressure after 72 hours of applied orthodontic force. However, effects on tooth movement were only observed with PTH concentration of 10 µg/100 g. Additionally, the authors compared the findings observed within 0, 3, 6, 9, and 12 days after the application of orthodontic force with subcutaneous injection of PTH 1-84 at 10 µg/100 g and PTH 1-34 at 0.4 and 4 µg/100 g; as well as dorsal injection of PTH-134 at 4 µg/100 g. The groups receiving PTH 1-84 at 10 µg/100 g and PTH 1-34 at 4 µg/100 g presented tooth movement rates approximately 3 times higher if compared with the control group 6, 9 and 12 days after the application of orthodontic force. On the other hand, even in the absence of significant tooth movement, the daily dorsal injection of PTH 1-34 at 4 µg/100 g revealed systemic alterations in the sample, such as increased bone mineral density, and phosphorus and alkaline phosphatase (ALP) serum levels (10).

In this way, Soma *et al.* (11) decreased the concentration and increased the release time of PTH combining the drug with methyl cellulose gel in local injections. Consequently, the authors observed that PTH with concentration level set at 1 µg/per animal presented significant improvement in tooth movement, within interspersed applications, if compared to any other group (11).

Yet Li *et al.* (12) used a higher concentration of PTH 1-34 (4 µg/100 g) and orthodontic force (40 g) in rats with lighter weight. Tooth movement was significantly higher 9 days after the application of orthodontic force. Additionally, the authors observed a major concentration of osteoclasts; tartrate-resistant acid phosphatase (TRAP) positive cells, receptor activator of nuclear factor kappa-B (RANKL); and insulin grown factor (IGF)-1 in periodontal region of pressure (12)

- Risk of bias across studies:

The scientific articles selected did not have compatible results that would allow a meta-analysis, since they were heterogenous. Furthermore, a meta-analysis could not be done due to lack of clinical studies on this topic.

## Discussion

Orthodontic tooth movement includes two main biological responses – bone resorption and formation (3,4). Interestingly the same degree of force, but with the opposite direction results in different effects on periodontal ligament cells. The bone region under pressure has predominant expression of pro inflammatory cytokines and chemokines, such as tumor necrosis factor (TNF)-α and Interleukin (IL)-1β (3). This physiological environment enables the activation of osteoclasts, which are mediators for the response of bone resorption. In fact, the predominant expression of pro inflammatory mediators directly increases the concentration of specific cell types, such as monocytes/macrogaphages, which precedes the osteoclast formation (3,15,16). Additionally, the presence of cytokines TNF-α and IL-1β are strictly associated with the expression of the main osteoclastogenic components – the RANK/RANKL system (15). Specifically, RANKL is linked to the receptor RANK in pre-osteoclastic (monocytes) cells generating intracellular signal through nuclear factor kappa B (NFkB) and AP-1; transcription factors for osteoclastic formation and activation; and consequent local bone resorption (17,18). Oppositely, the bone region under tension reveals an anti-inflammatory environment, with prevalent expression of anti-inflammatory cytokines, such as IL-10; and transforming growth factor (TGF)-β (3). IL-10 and TGF-β negatively interfere within the expression of TNF-α and IL-1β; increases the expression of osteoprotegerine (OPG) – a decoy of RANK that decreases the RANK/RANKL binding; and consequently avoids the osteoclasts formation (15,17,18).

Once the orthodontic movement presents two completely different environments, the discovery and/or administration of a drug that accelerates or increase the effectiveness of bone resorption and formation at the same time becomes extremely difficult. In fact, a systemic drug which increases the rate of bone resorption by osteoclasts, possibly interfere negatively on bone formation by osteoblasts on the opposite side of the tooth. Similarly, agents that promote bone formation possibly reduce the handling time for interfering with the absorption area of the pressure side.

Teriparatide arose as a systemic synthetic drug with the same amino acid sequence of the PTH active form, which is currently used as long-term treatment option for patients with osteoporosis; and this drug can interfere in a positive way in both sides (pressure and tension area). In the tension area, Teriparatide is linked to the PTH receptor identified in osteoblasts and related prece-ding cells – pluripotent progenitors of mesenchymal lineage (19). Thus, the main intracellular mechanism of activity of Teriparatide in the osteoblasts occurs from its interaction with the specific receptor type I (PRPI), which is attached to the G protein (8). In the bone region under tension, anabolic bone effects are observed. Mainly, these effects are associated with the link between Teriparatide and PRPI attached to Gq protein, which activates kinase C, controlling the formation of growth factors (IGF-I, IGF-II e TGF-β) and indirectly increasing the expression of OPG (inhibiting osteoclast formation). Optionally, Teriparatide may also link with PRPI receptor and stimulate adenilciclase in osteoblasts (main structures for RANKL production), increasing the concentration of cyclic AMP and activating protein-kinase A (PKA) associated to the secretion of IL-6 and de RANKL – chain factors for bone resorption (8). Thus, Teriparatide may enhance bone turnover and interferes within the time for orthodontic tooth movement maintaining quality after bone formation response. Based on that, Teriparatide may potentially optimize both the bone response for resorption and formation (2,8,16).

Despite the apparent benefits of Teriparatide, effective tooth movement depends on the type of drug administration and concentration (10,12). Specifically in orthodontics, direct effect of Teriparatide in osteoblasts is expected. Based on that, local drug injection is the best approach once systemic administration may spread the drug to different body region decreasing the drug concentration in the bone adjacent to the teeth under movement. This is observed administrating subcutaneous PTH at 4 µg/100 g on the back region of Wistar rats and comparing with controlled rats under orthodontic treatment (10). Oppositely, a higher dose of PTH (10 µg/100 g) improved the tooth movement if compared with the control group (10). It may indicate that higher drug concentration within systemic administration provides major remaining dose in the bone adjacent to the teeth under movement (10). Additionally, the higher drug dose increased the number of osteoclasts in region under pressure and decreased the time for bone resorption, avoiding local necrosis (10).

Apparently, PTH must be chronically administrated for a proper effectiveness within tooth movement, following local application with ideal quantity. Several other drugs that require similar conditions were tested for application diluted within drug carriers and other substances to increase the drug half-life in the anatomic site of interest. In fact, the local and systemic administration of PTH with concentration of 1 µg/400 g did not result with statistically significant tooth movement (11). However, the same local (periodontal region) drug set up diluted within MC increased the tooth movement rate 2x if compared to the group of receiving PTH diluted in saline (11). Methyl-cellulose gel is already used combined with lidocaine to enhance nerve blocking during anesthetic interventions, without revealing side effects (20). Based on that, this gel has the capacity to decrease the drug release in the anatomic site of application, making it available for a longer period within low quantity, which is an ideal condition for the chronic administration of PTH aiming tooth movement (8).

Local concentration and the period of drug released are apparently important factors affecting its effectiveness in tooth movement. Additionally, the size of the protein also seems to influence the outcomes. Despite the fact that Teriparatide has the same effect of PTH (with 34 active amino acids present in the hormone), it has a smaller structure, which may present differences found within the experimental models of orthodontic movement, causing confusion and divergence in the current literature. The administration of systemic PTH with concentration of 4 µg/100 g on a daily basis, in rats weighting 350-400 g, resulted within an increasing amount of serum markers for bone apposition and mineral bone density, but not on tooth movement acceleration (10). Yet the Teriparatide systemically administrated with concentration of 4 µg/100 g every other day, in rats weighting approximately 200g, resulted within improved tooth movement and increased amount of RANKL+ and TRAP+ cells in the bone region under pressure, as well as higher positivity for OPG and IGF-1 when compared to control groups, indicating a high expression of osteoclastogenic and bone formation factors. Once Teriparatide is structurally smaller than PTH, it may maintain a higher concentration in the periodontal region or even major in vivo affinity with PTH receptors in the osteoblasts of the periodontal ligament. Further studies on the affinity to PTH receptors and its activation are encouraged considering that Teriparatide and PTH may present a different activation of PTK, triggering different intracellular signaling with opposite effects.

Despite complex and paradoxical, the administration of Teriparatide and even PTH in experiments involving induced tooth movement in rats may influence on tooth movement acceleration depending on the drug administration (local/systemic) and time of release, concentration and periodicity (10-12). Evidently, additional researches remain necessary to elucidate the mechanism of Teriparatide interaction with bone apposition and resorption within tooth movement in humans. However, considering the chronic administration of Teriparatide in low concentration doses in patients with osteoporosis, and even the individual genetic profile and pathologies, which cause different concentrations on the physiological production of PTH production, orthodontic patients may present different responses to applied forces, requiring major attention of the Orthodontists in face of the patient’s clinical status. Additionally, Teriparatide revealed to be a potential option for further studies attempting to improve the clinical routine of orthodontic mechanics.

In this way, this systematic review concluded that there is evidence showing that the use of teriparatide may accelerate the induced tooth movement in rats depending on the way of drug administration and the time for drug release.

## References

[1] Braga SM, Taddei SR, Andrade I, Queiroz-Junior CM, Garlet GP, Repeke CE (2011). Effect of diabetes on orthodontic tooth movement in a mouse model. Eur J Oral Sci.

[2] Jiang C, Li Z, Quan H, Xiao L, Zhao J, Jiang C (2015). Osteoimmunology in orthodontic tooth movement. Oral Dis.

[3] Garlet TP, Coelho U, Silva JS, Garlet GP (2007). Cytokine expression pattern in compression and tension sides of the periodontal ligament during orthodontic tooth movement in humans. Eur J Oral Sci.

[4] Garlet TP, Coelho U, Repeke CE, Silva JS, Cunha FQ, Garlet GP (2008). Differential expression of osteoblast and osteoclast chemmoatractants in compression and tension sides during orthodontic movement. Cytokine.

[5] Pietschmann P, Mechtcheriakova D, Meshcheryakova A, Foger-Samwald U, Ellinger I (2016). Immunology of osteoporosis: a mini-review. Gerontology.

[6] Plotkin H, Gundberg C, Mitnick M, Stewart AF (1998). Dissociation of bone formation from resorption during 2-week treatment with human parathyroid hormone-related peptide-(1-36) in humans: potential as an anabolic therapy for osteoporosis. J Clin Endocrinol Metab.

[7] Dobnig H (2004). A review of teriparatide and its clinical efficacy in the treatment of osteoporosis. Expert Opin Pharmacother.

[8] Kraenzlin ME, Meier C (2011). Parathyroid hormone analogues in the treatment of osteoporosis. Nat Rev Endocrinol.

[9] Thiruchelvam N, Randhawa J, Sadiek H, Kistangari G (2014). Teriparatide induced delayed persistent hypercalcemia. Case Rep Endocrinol.

[10] Soma S, Iwamoto M, Higuchi Y, Kurisu K (1999). Effects of continuous infusion of PTH on experimental tooth movement in rats. J Bone Miner Res.

[11] Soma S, Matsumoto S, Higuchi Y, Takano-Yamamoto T, Yamashita K, Kurisu K (2000). Local and chronic application of PTH accelerates tooth movement in rats. J Dent Res.

[12] Li F, Li G, Hu H, Liu R, Chen J, Zou S (2013). Effect of parathyroid hormone on experimental tooth movement in rats. Am J Orthod Dentofacial Orthop.

[13] Liberati A, Altman DG, Tetzlaff J, Mulrow C, Gotzsche PC, Ioannidis JPA (2009). The PRISMA statement for reporting systematic reviews and meta-analyses of studies that evaluate health care interventions: explanation and elaboration. PLoS Med.

[14] Cericato GO, Bittencourt MA, Paranhos LR (2015). Validity of the assessment method of skeletal maturation by cervical vertebrae: a systematic review and meta-analysis. Dentomaxillofac Radiol.

[15] Yamaguchi M (2009). RANK/RANKL/OPG during orthodontic tooth movement. Orthod Craniofac Res.

[16] Goltzman D, Hendy GN (2015). The calcium-sensing receptor in bone--mechanistic and therapeutic insights. Nat Rev Endocrinol.

[17] Nakashima T, Takayanagi H (2009). Osteoimmunology: crosstalk between the immune and bone systems. J Clin Immunol.

[18] Takayanagi H (2012). New developments in osteoimmunology. Nat Rev Rheumatol.

[19] Swarthout JT, D'Alonzo RC, Selvamurugan N, Partridge NC (2002). Parathyroid hormone-dependent signaling pathways regulating genes in bone cells. Gene.

[20] Paavola A, Yliruusi J, Kajimoto Y, Kalso E, Wahlstrom T, Rosenberg P (1995). Controlled release of lidocaine from injectable gels and efficacy in rat sciatic nerve block. Pharm Res.

